# Tuberculosis in Washington

**Published:** 1906-08

**Authors:** 


### Abstracts and Selections

TUBERCULOSIS IN WASHINGTON.—Gov. A. E. Mead, of Washington, one of the honorary vice-presidents of the American International Congress on Tuberculosis, is actively interested in the campaign against consumption and is fully alive to the necessity of restrictive legislation. He writes me: "I will do my utmost to carry on the work of the Congress, and to promote its purposes in any way I can." He will send a large delegation from his State to the New York meeting in November next. I extract the following from his inaugural address as Governor of Washington:

*Tuberculosis:* Our State is entitled to special prominence, when compared with any other State in the Union, as a country possessing a health-giving climate. Its abundance of fresh water for domestic use, its bracing air, untainted by the poison of malaria, are great factors in lessening the progress of disease. It does not, however, possess the temperature and other conditions needed by those seeking relief from pulmonary troubles that may be found in southern climes. It is estimated by a writer in one of the current magazines that of the 75,000,000 living Americans, 8,000,000 must inevitably die from pulmonary tuberculosis. Necessarily we must share in the terrible levy made annually upon the lives of the people. Can we afford to be dilatory in aligning our State with the progressive States of the Union in declaring war upon the great white plague, which is, in fact, the scourge of the world? I ask you, therefore, to strengthen the powers of our local and State boards of health, arming them with weapons to give battle to this arch enemy of mankind.

Let me fortify this recommendation with an extract from the fifth biennial report of our State Board of Health, which is as follows:

"Tuberculosis in this State, as in all other States, is the cause of more deaths than any one other disease. It is settled beyond doubt that it is a communicable disease and one which, with reasonable precautions, can be avoided. Notwithstanding these facts, little or no precaution is taken by the people generally, though boards of health and physicians have for years been endeavoring to educate the public to the dangers and how to avoid them."

